# Low Soluble Receptor for Advanced Glycation End Products Precedes and Predicts Cardiometabolic Events in Women With Rheumatoid Arthritis

**DOI:** 10.3389/fmed.2020.594622

**Published:** 2021-01-28

**Authors:** Mitra Nadali, Lovisa Lyngfelt, Malin C. Erlandsson, Sofia Töyrä Silfverswärd, Karin M. E. Andersson, Maria I. Bokarewa, Rille Pullerits

**Affiliations:** ^1^Department of Rheumatology and Inflammation Research, Institution of Medicine, Sahlgrenska Academy at University of Gothenburg, Gothenburg, Sweden; ^2^Rheumatology Clinic, Sahlgrenska University Hospital, Gothenburg, Sweden; ^3^Department of Clinical Immunology and Transfusion Medicine, Sahlgrenska University Hospital, Gothenburg, Sweden

**Keywords:** advanced glycation end product, cardiovascular disease, soluble RAGE, rheumatoid arthritis, cardiometabolic events

## Abstract

**Background:** Cardiovascular disease (CVD) causes premature mortality in rheumatoid arthritis (RA). Levels of soluble (s)RAGE change with aging, hypertension and hypercholesterolemia. We assessed whether sRAGE was associated with increased risk of CVD in RA patients.

**Methods:** Serum sRAGE was measured in 184 female RA patients and analyzed with respect to CVD risk estimated by the Framingham algorithm (eCVR), metabolic profile and inflammation. Levels of sRAGE in 13 patients with known cardio-metabolic morbidity defined the cut-off for low sRAGE. Prospective 5-year follow-up of new CV and metabolic events was completed.

**Results:** Low sRAGE was significantly associated with previous history and with new imminent cardiometabolic events in the prospective follow-up of RA patients. In both cases, low sRAGE reflected higher estimation of CVR in those patients. Low sRAGE was attributed to adverse metabolic parameters including high fasting plasma glucose and body fat content rather than inflammation. The association of sRAGE and poor metabolic profile was prominent in patients younger than 50 years.

**Conclusions:** This study points at low sRAGE as a marker of metabolic failure developed during chronic inflammation. It highlights the importance for monitoring metabolic health in female RA patients for timely prevention of CVD.

**Trial registration:** ClinicalTrials.gov with ID NCT03449589. Registered 28, February 2018.

## Introduction

Glycation is the process of non-enzymatic binding of sugar molecules glucose and fructose with proteins, lipids and nucleic acids. Glycation directly depends on glucose concentration and occurs at random sites of a molecule. It leads to the loss of molecule's function and degradation into the advanced glycation end products (AGEs) (1). Excessive glycation may occur both in response to the oxidative stress, hypoxia and inflammation. In turn, circulating AGEs in the extracellular compartment activate the proinflammatory receptor for advanced glycation end products (RAGE) and participate in perpetuation of inflammation (2). Under inflammatory conditions, other non-glycated RAGE ligands such as S100 proteins and HMGB1 are accumulated. RAGE ligands induce proinflammatory signaling through the membrane-bound RAGE causing nuclear translocation of NF-kappa B followed by cytokine production (2).

Broad range of harmful consequences of long lasting hyperglycosemia for health is well-documented (3), while cellular malfunction in response to high circulating glucose requires better understanding. Exposure of proteins to glucose enhances the process of unselective glycation (4–6). Measurement of glycated hemoglobin is clinically used to monitor DM (4). Ingestion of high glycated milk protein results in a rise of plasma glucose (7). However, there is a controversial view on levels of sRAGE in T2D. Several studies indicated decreased levels of sRAGE in T2D without complications (8, 9) and others reported high levels of sRAGE in T2D with cardiovascular or renal complications due to increased production of AGEs (10–12). AGEs have a key role in chronic inflammation and their accumulation has reported both in CVD, atherosclerosis and RA. Other factors as male gender, smoking and hyperglycemia have been reported to raise generation of AGEs independently to RA. Interestingly disease activity or erosivity of RA had no association with AGEs (13).

RAGE is a multiligand receptor, which belongs to the immunoglobulin superfamily of cell surface molecules and is physiologically expressed by cells involved in innate immune responses, including macrophages and granulocytes, and also on endothelial cells, vascular smooth muscle cells, and adipocytes (14). A soluble form of RAGE (sRAGE) is either generated via the proteolytic cleavage of extracellular domain of the membrane-bound RAGE or formed by endogenous splicing of RAGE mRNA transcripts. It acts as a decoy receptor by catching RAGE ligands and preventing them from binding to the membrane-bound RAGE and thereby modulating the pro-inflammatory effects of RAGE signaling (2, 15). Soluble RAGE is considered to protect against adverse effects of proinflammatory RAGE ligands. Low levels of sRAGE were suggested to be a very early marker of endothelial dysfunction (16), and were reported in coronary artery disease (17, 18), atherosclerosis (19), essential hypertension (20, 21), hypercholesterolemia (22), and in RA (23), where CVD remained to be the major cause of premature death. We have previously reported that chronic inflammation in RA is associated with significantly lower serum sRAGE compared to healthy controls and patients with non-inflammatory joint diseases (23). Furthermore, the presence of anti-RAGE antibodies locally in the joints of RA patients was related to a less destructive joint disease (24).

In the present prospective study, we assess an association between serum sRAGE and cardiometabolic health in female RA patients. We search for the CVD risk factors attributed to the low serum levels of sRAGE.

## Materials and Methods

### Patients

One hundred eighty-four female patients with established RA were recruited into the study. All the patients fulfilled the American Rheumatism Association 1987 revised criteria for RA (25). Patients were randomly chosen from the methotrexate (MTX)-treated patient cohorts at two rheumatology units in Sweden, Sahlgrenska University Hospital in Gothenburg and the Northern Älvsborg Country Hospital in Uddevalla during the period from November 2011 until September 2013. Patients under the age of 18, patients with other rheumatologic diseases, and juvenile idiopathic arthritis were excluded. At inclusion, 93% (*n* = 172) of patients received MTX treatment. Fifty-one patients (28%) had treatment with biologics including infliximab (*n* = 23), etanercept (*n* = 12), golimumab (*n* = 5), adalimumab (*n* = 3), rituximab (*n* = 3), tocilizumab (*n* = 4), abatacept (*n* = 1). Twenty-five MTX-treated patients (16%) received concomitantly other disease modifying drugs (14 sulfasalazine, 6 hydroxychloroquine, 4 combination of sulfasalazine and hydroxychloroquine, and 1 cyclosporine A). Oral corticosteroids (median dose 5.0 mg/day) were regularly used by 20 patients (11%). All patients completed the questionnaire about their current medication, concomitant diseases and smoking habits. At inclusion, all patients were examined by experienced rheumatologists and the clinical (tenderness and swelling of 28 joints) and laboratory (erythrocyte sedimentation rate, C-reactive protein) disease activity variables were recorded. Disease activity score in 28 joints (DAS28) was calculated (http://www.4s-dawn.com/DAS28/). The clinical information with regard to patients' age, sex, body mass index (BMI), body fat content (26), and disease duration were collected.

### Ethical Consideration

The study was approved by the Swedish Ethical Review Authority (Dnr. 659-2011) and performed in accordance with the Declaration of Helsinki. The informed written consent was obtained from all subjects prior to enrolment in the study. The trial is registered at ClinicalTrials.gov with ID NCT03449589.

### Calculation of Estimated Cardiovascular Risk

A 10-year risk for development of CVD was estimated (eCVR) using a digital version of the Framingham algorithm (27) and included sex, age, systolic blood pressure, treatment for hypertension, current smoking, diabetes, HDL, and total cholesterol.

### CVD Follow-Up at 5 Years

Five years after enrollment, the patients were contacted for a structured telephone interview and a questionnaire was sent to their home address. The questions were asked for any CV event, and about current medication with antihypertensive drugs, anticoagulants, anti-diabetic drugs, and use of statins. The reported CV events and changes in medications were then controlled in patients' medical records and the Swedish National Health Registry. We were able to reach all patients except 3 patients—two of them were diseased and one patient had moved out of Sweden.

### Collection and Preparation of Blood Samples

The blood samples were obtained after overnight fast. Blood was collected from the peripheral cubital vein directly into the vacuum tubes containing serum clot activator (Vacuette, Greiner Bio-One, Kremsmunster, Austria), mixed thoroughly and left to coagulate for 3–4 h at room temperature. The tubes were then centrifuged at 2,000 × g for 10 min, the serum carefully collected, aliquoted, and stored at −80°C until use.

### Measurement of sRAGE

The levels of sRAGE in serum were determined using a specific sandwich ELISA kit (R&D Systems, Minneapolis, MN, USA) according to the manufacturer's protocol. Serum was diluted 1/3 in assay buffer and introduced into the ELISA plates coated with mouse monoclonal antibody against RAGE. After 2 h of incubation with serum, polyclonal capture antibody against the extracellular portion of RAGE was used. The reaction was visualized by tetramethylbenzidine substrate. The minimum detectable concentration of sRAGE was 4 pg/ml. According to the manufacturer, no significant cross-reactivity to EN-RAGE, HMGB1, S100A10, or S100Baa was observed.

### Other Serological Measures

The measurement of adipokines and cytokines were determined using specific sandwich ELISA kits according to the instructions from the manufacturers (R&D Systems, Minneapolis, MN, USA) as previously described (28). The inflammatory parameters, blood lipids and RF/ACPA antibodies were measured at the accredited Laboratory of Clinical Chemistry at the Sahlgrenska University Hospital according to clinical routines. Plasma glucose levels were measured using FreeStyle Lite kit (Abbott Diabetes Care Ltd., Oxon, UK) and insulin levels by sandwich ELISA kit (DY8056, R&D Systems, Minneapolis, MN, USA).

### Statistical Analysis

Descriptive statistics for continuous variables are presented as the median with interquartile range, and for categorical variables as the number and the percentage. Univariate correlation between variables was examined by the Spearman's correlation test. Any two factors with a correlation coefficient >0.3 were investigated for co-linearity. For continuous variables, the difference between groups was assessed by using the Mann-Whitney *U*-test. The difference in frequency, sensitivity and specificity of calculations were performed using Chi Square and Fisher's exact test. Analyses were performed using Graph Pad Prism 8 for Microsoft Windows. All tests were two tailed and *p* < 0.05 was considered statistically significant.

## Results

### Soluble RAGE and Clinical, Metabolic, and Inflammatory Features in RA

Out of 184 patients included at the baseline, we identified 7 patients with type 2 diabetes (T2D) and 6 patients with previous CV events. As T2D and CVD could affect sRAGE levels, these 13 patients with cardiometabolic diseases were extracted from the cohort, analyzed separately and comprised a cardiometabolic reference (CMR) group. The baseline characteristics of CMR group (*n* = 13) and the remaining study cohort (*n* = 171) are shown in Table 1.

**Table 1:** The baseline characteristic of the study cohort (*n* = 171), female patients with RA.

	**RA study cohort**	**CMRG**
	***n* = 171**	***n* = 13**
sRAGE, pg/ml	1,417 [1,093–1738]	1,259 [1,114–1,504]
Age, years	53 [44–62]	60 [53–63]
Disease duration, years	7 [4–14]	8 [3.5–19]
BMI, kg/m^2^	24.85 [22.3–28]	27 [25.7–29.4]
Body fat, %	36.32 [32.37–41.7]	39.4 [36.6–43.6]
TG/HDL, ratio	0.5 [0.3–0.7]	3.1 [2.4–4.0]
DAS-28	3.0 [2.3–3.9]	3.5 [3.0–4.6]
ESR, mm/h	9 [5–14]	11 [6–15]
RF and/or ACPA positive	157/170 (92%)	13/13 (100%)
Methotrexate dose, mg/week	17.5 [12.5–20]	17.5 [5.6–24]
Biological DMARD	51(30%)	4/13 (31%)
TNFα-inhibitors	43 (25.1%)	1/13 (7.6%)
Medication for hypertension	22 (13%)	6/13 (46%)
Medication for dyslipidemia	9 (5.2%)	1/13 (15%)
Current medication with NSAID	44 (26%)	2/13 (15%)
Current oral corticosteroids,	20/171 (12%)	1/13 (7.6%)
Current oral corticosteroids, mg/day	5 [5–5]	1/13 (7.6%)
eCVR, %	6.6 [3.8–11.0]	18 [7.5–29.2]
SBP, mmHg	130 [120–140]	135 [123–140]
Current smoker	23/170 (13%)	3/13 (23%)
Former smoker	96/170 (57%)	10/13 (77%)
IL6, pg/ml	2.26 [0.12–8.94]	2.53 [1.48–7.67]
IL1β pg/ml	0 [0–10.6]	0 [0–0]
Leptin/adiponectin ratio	4.0 [1.6–10]	7.7 [1.7–28]
Resistin, ng/ml	21 [13–37]	21 [7.0–42]
Visfatin, ng/ml	2.58 [1.05–4.58]	2.4 [1.0–7.0]
IGF1, μg/l	138 [109–176]	139 [104–188]

The RA patients with diagnosed cardiometabolic diseases/events (n = 13) are referred to as cardiometabolic reference group (CMRG). The data are expressed as median [interquartile range] and number (%).

*ACPA, anti-citrullinated protein antibody; bDMARD, biological disease modifying anti rheumatic drug; BMI, body mass index; eCVR, estimated cardiovascular risk; ESR, estimated sedimentation rate; HDL, high density lipoprotein; IGF1, insulin-like growth factor 1; IL, interleukin; MTX, methotrexate; NSAID, none steroidal anti-inflammatory drug; RF, rheumatoid factor; SBP, systolic blood pressure; TG, triglyceride; RTX, rituximab; sRAGE, soluble receptor for advanced glycation end products*.

Expectedly, CMR group had significantly higher eCVR compared to the remaining 171 patients (Table 1). This high CVR was largely attributed to high fasting plasma glucose levels and adverse composition of blood lipids including TG and TG/HDL ratio, leptin/adiponectin ratio, and BMI (Table 1). CMR group had significantly higher disease activity estimated by DAS28 compared to the remaining 171 RA patients. Interestingly, sRAGE levels had significant strong positive correlation with insulin (*r* 0.643, *p* = 0.028), HOMA index (*r* 0.626, *p* = 0.032), and age (*r* 0.675, *p* = 0.013) within the CMR group.

**Figure 1:**
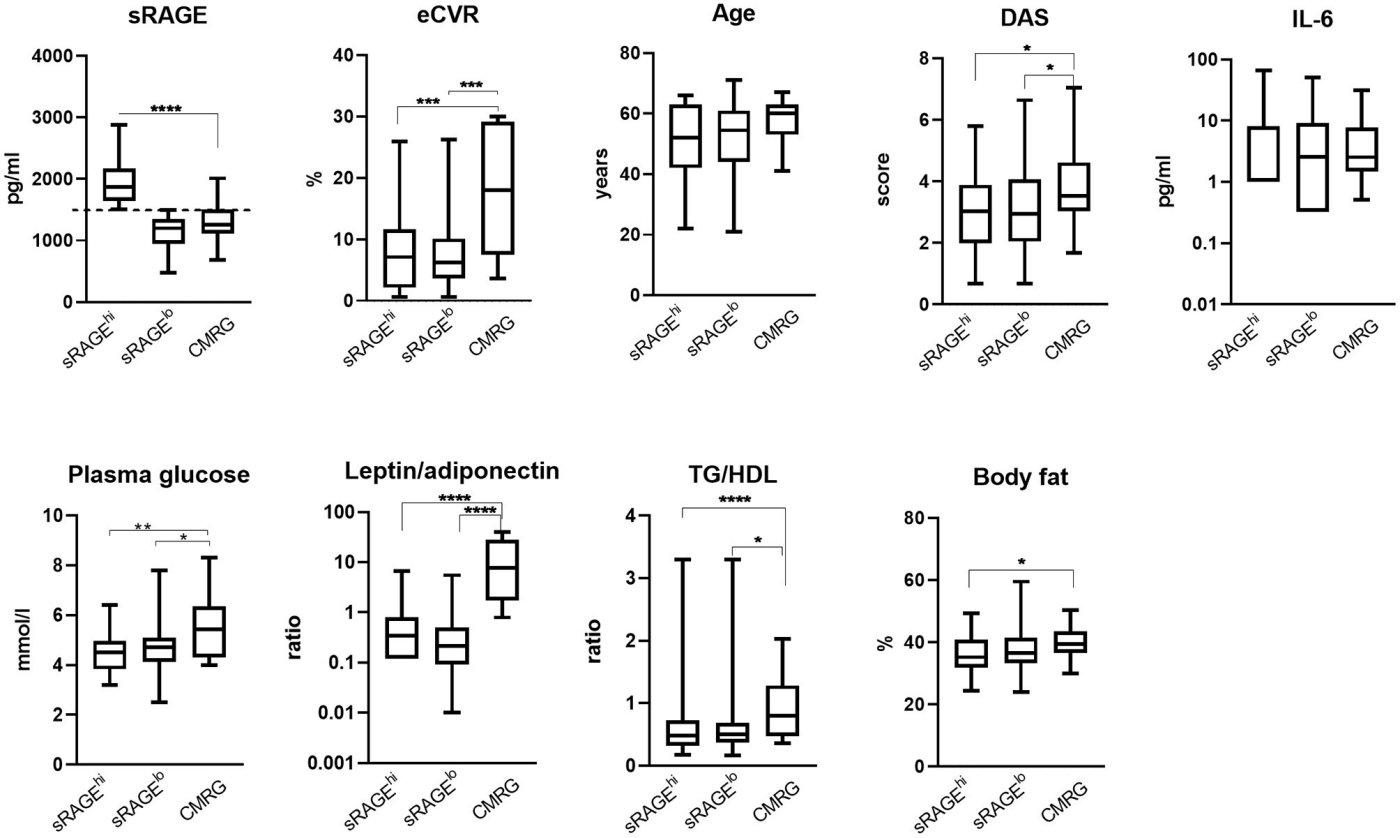
Metabolic and inflammation-related characteristics of female patients with rheumatoid arthritis. Serum levels of soluble receptor for advanced glycation end products (sRAGE) were measured in 184 patients and the patients with no previous cardiovascular events were divided into the high (sRAGE^hi^, *n* = 73) or low (sRAGE^lo^, *n* = 98) groups, accordingly. Cardio-metabolic reference group (CMRG) consisted of patients with history of cardiovascular events and/or type II diabetes (*n* = 13). SRAGE in the upper quartile of CMR group (above 1,504 pg/ml, indicated with dotted line) defined the cut-point for dichotomization. Statistical evaluations were calculated using the Mann-Whitney *U*-test. The box plots indicate medians and interquartile ranges; whiskers show min to max. *P < 0.05; **P < 0.01; ***P < 0.001; and ****P < 0.0001. TG/HDL, ratio between serum triglycerides and high-density lipoproteins; DAS28, disease activity score; IL, interleukin.

To investigate whether sRAGE concentrations were associated with high CV risk, sRAGE values within the lower 75% of the CMRG were considered low and were used to dichotomize the CV event free RA patients into high sRAGE (sRAGE^hi^; *n* = 73) and low sRAGE (sRAGE^lo^; *n* = 98) groups (Figure 1). The median eCVR was comparable between the groups with high and low sRAGE levels (Figure 1). We found neither differences in cardiometabolic nor in RA-related disease activity parameters (Figure 1).

**Figure 2:**
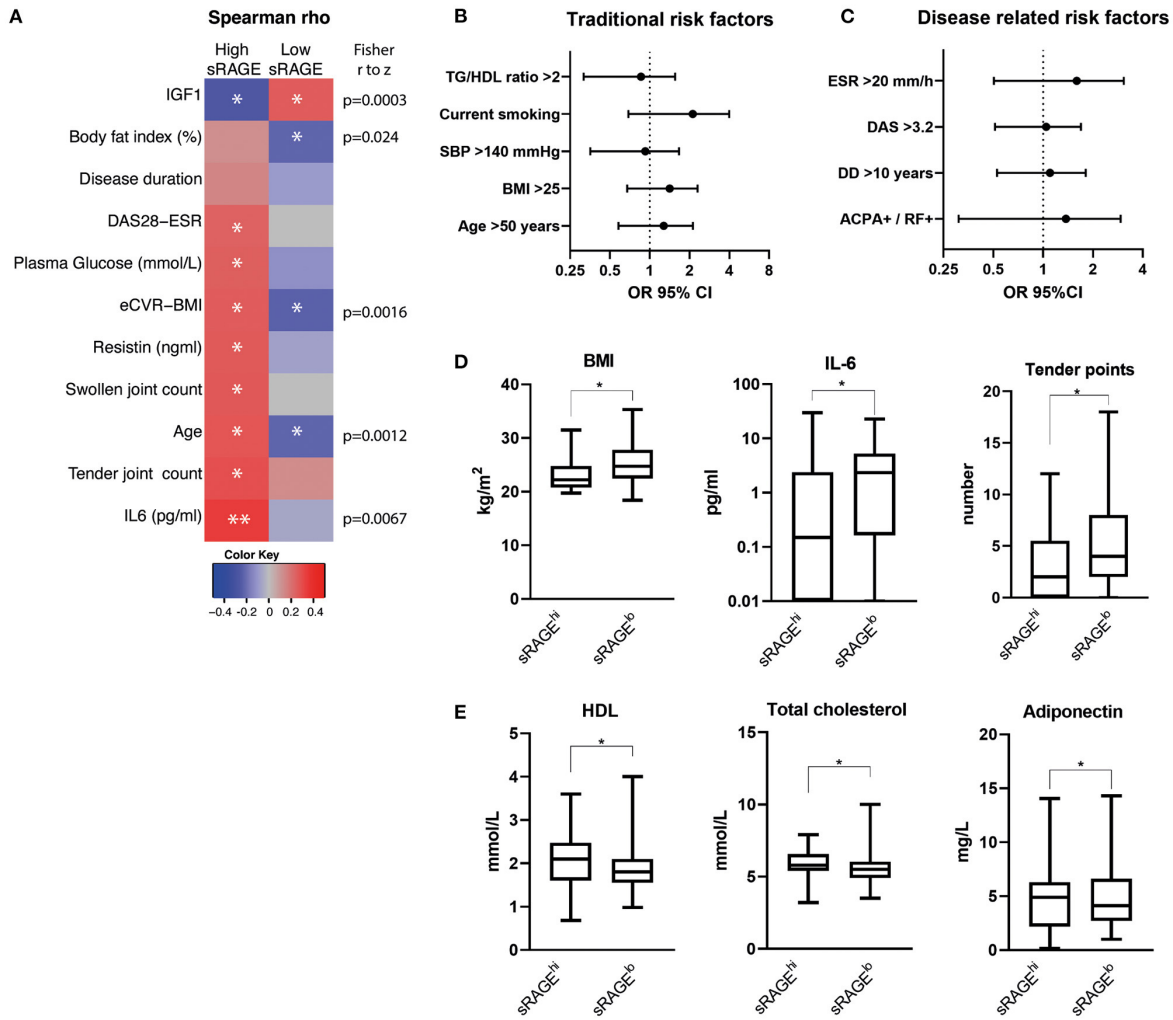
Correlation between sRAGE levels and cardio-metabolic and inflammatory parameters within respective groups **(A)** and the differences in frequency of the traditional cardiovascular risk factors **(B)** and RA-related risk factors **(C)** between the patients with high (*n* = 73) and low (*n* = 98) serum levels of soluble RAGE. Correlation matrix in **(A)** shows Spearman's *R*-values in color as indicated by color key code. *P< 0.05; **P < 0.01. The differences as indicated by forest plots in **(B,C)** are calculated as odds ratio (OR) with 95% confidence interval (CI). The *p*-values are obtained by chi-square statistics. Comparison of the traditional, inflammation- and RA-related CVR factors for ages <50 years (*n* = 66) **(D)**, and ≥50 years (*n* = 105) **(E)**, in sRAGE^lo^ and sRAGE^hi^ group. The comparison is done pairwise using the Mann-Whitney *U*-test. The box plots indicate medians and interquartile ranges; whiskers show min to max. *P < 0.05; **P < 0.01. BMI, body mass index; DAS28, disease activity score with assessment of 28 joints; eCVR, estimated cardiovascular risk; IL, interleukin; RAGE, receptor for advanced glycation end products; HDL, high density lipoprotein. TG/HDL, ratio between triglycerides and high-density lipoprotein; TC, total cholesterol; ESR, erythrocyte sedimentation rate; ACPA/RF, presence of antibodies to citrullinated peptide antibodies and/or rheumatoid factor; DD, disease duration; SBP, systolic blood pressure.

Next, we performed univariate correlation analysis between sRAGE and CV risk parameters in un-dichotomized RA cohort and observed bi-directional correlation profile between sRAGE and eCVR (Supplementary Figure 1). Thus, we analyzed correlation between sRAGE levels and cardio-metabolic and inflammatory parameters within respective group. The correlation pattern of sRAGE was remarkably different between the sRAGE^hi^ and sRAGE^lo^ patients (Figure 2A). This difference in correlation between RAGE^hi^ and sRAGE^lo^ groups was confirmed by the Fisher r-to-z test and was significant for eCVR-BMI, body fat index, age, IL-6, and IGF-1 (Figure 2A). Additionally, in patients within sRAGE^hi^ group, sRAGE showed significant positive correlation with plasma glucose, eCVR and age. In relation to RA-related risk factors, sRAGE correlated positively with DAS28, tender and swollen joints, IL6, and resistin, whereas a negative correlation was seen between sRAGE and serum levels of IGF1 (Figure 2A). In contrast, in patients within sRAGE^lo^ group, a positive correlation was seen between sRAGE level and IGF1, whereas eCVR-BMI and body fat content correlated negatively to sRAGE.

The analysis of traditional CVR factors such as hypertension, dyslipidemia, overweight, smoking, and age showed no significant difference between sRAGE^lo^ and sRAGE^hi^ groups (Figure 2B).

We thereafter studied RA-related CVR factors, which included higher ESR, the presence of RA-specific antibodies, long disease duration and active RA disease defined by DAS28. The comparison showed that none of the RA-related risk factors had significant difference between sRAGE^lo^ and sRAGE^hi^ groups (Figure 2C).

Since eCVR is age dependent and a decrease of sRAGE levels with increasing age has been reported in several studies (29–31), we performed the analysis separately for the patients of different age groups. We compared the traditional and RA-related CVR factors as well as serum levels of adipokines and cytokines for ages <50 years (*n* = 66), and ≥50 years (*n* = 105) in sRAGE^lo^ and sRAGE^hi^ group. We observed no significant differences in sRAGE levels between the patients <50 years compared to those ≥50 years within respective sRAGE^lo^ and sRAGE^hi^ groups.

We found that dominating CVR parameters in sRAGE^lo^ group were age dependent. In RA patients <50 years, low sRAGE group (*n* = 37) was significantly different from high sRAGE group (*n* = 29) by having higher BMI (*p* = 0.028), IL-6 concentration (*p* = 0.019), and more tender points (*p* = 0.042) (Figure 2D). Thus, metabolic and inflammation-related factors dominated CVR in patients <50 years. In RA patients ≥50 years, low sRAGE group (*n* = 61) showed significant differences in the profile of blood lipids. We found significantly lower HDL (*p* = 0.023), lower total cholesterol (*p* = 0.018), and adiponectin (*p* = 0.023), compared to high sRAGE group (*n* = 44) (Figure 2E).

### Prospective Follow-Up for Development of New Cardiometabolic Events

Within 5 years, 11 of 171 patients (6.4%) developed new cardiometabolic events (CME). In the sRAGE^lo^ group, seven events were observed including 1 patient with new T2D diagnosis, 2 chronic atrial fibrillations (AF), 2 transitory ischemic attacks, 1 patient got deep venous thrombosis and one patient deceased due to aorta dissection. In the sRAGE^hi^ group, CME occurred in 4 patients including 1 patient with new T2D combined with AF, and 1 AF, 1 stroke, and 1 incidental aortic aneurysm were reported. The prevalence of new CME was not different between the sRAGE^lo^ and sRAGE^hi^ groups (6.9 vs. 6.1%, respectively).

**Figure 3:**
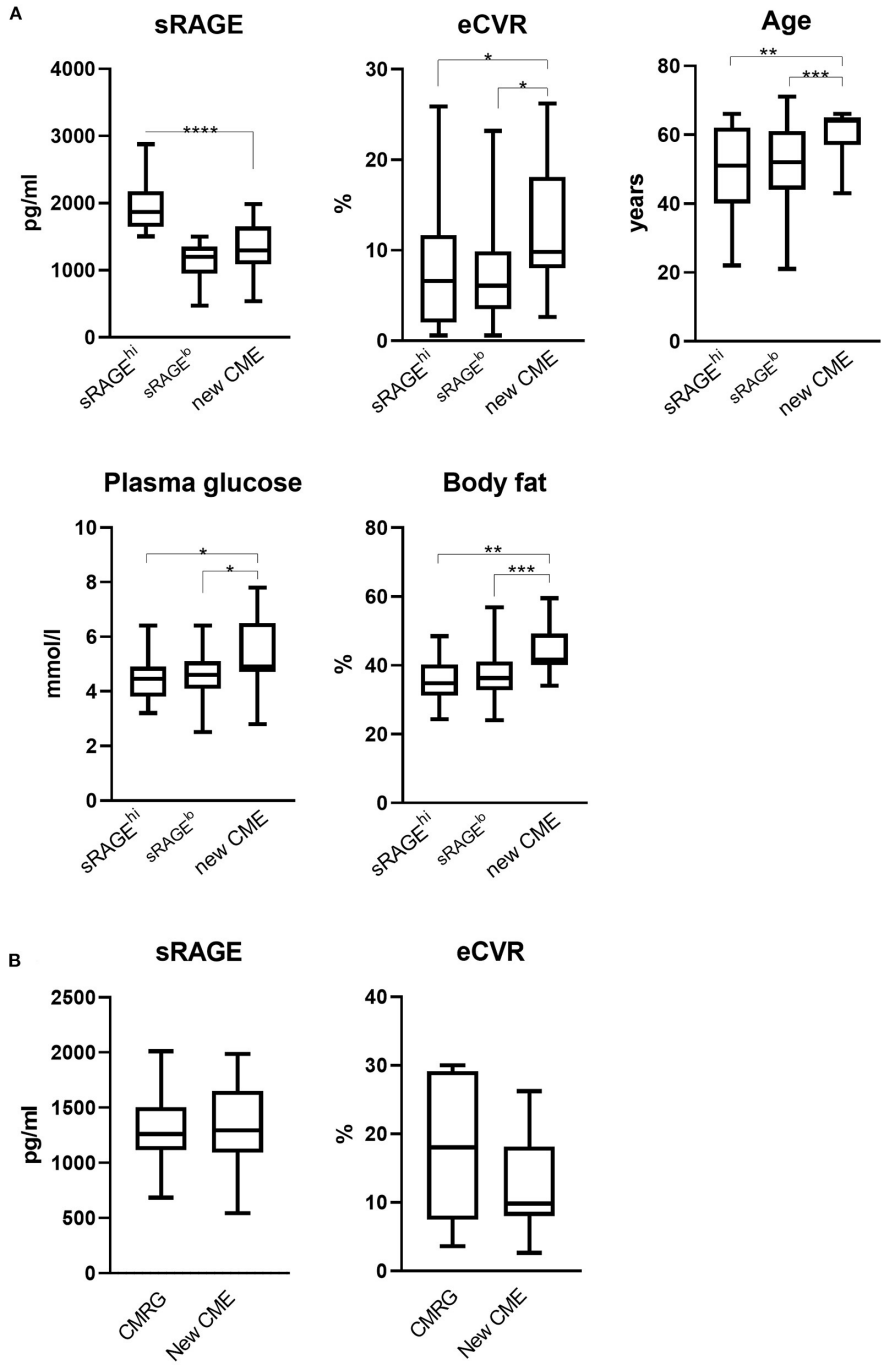
**(A)** Comparison of metabolic and inflammation-related characteristics of RA patients with new cardiometabolic events. During the prospective follow up for 5 years, 11 of 171 patients developed new cardiometabolic events (CME). This group was compared to the patients with high (sRAGE^hi^, *n* = 69) and low (sRAGE^lo^, *n* = 91) serum levels of soluble RAGE. eCVR, estimated cardiovascular risk; RAGE, receptor for advanced glycation end products. **(B)** Comparison of new CME group (*n* = 13) with CMR (*n* = 11) group with respect to eCVR and sRAGE. *The comparison is done pairwise using the Mann-Whitney *U*-test. The box plots indicate medians and interquartile ranges; whiskers show min to max. *P < 0.05; **P < 0.01; ***P < 0.001; and ****P < 0.0001.

Next, we wanted to study whether the patients with new CME were different at inclusion with respect to inflammation and metabolic characteristics compared to patients in sRAGE^hi^ and sRAGE^lo^ groups that had no new CME. The new CME group had significantly lower sRAGE levels compared with the sRAGE^hi^ group (Figure 3A). Importantly, new CME group had significantly higher eCVR compared to both the sRAGE^lo^ and sRAGE^hi^ groups. Patients in the new CME group were significantly older and had the adverse metabolic parameters such as higher plasma glucose levels and increased body fat compared with patients in the sRAGE^hi^ and sRAGE^lo^ groups. Inflammation, measured by ESR, IL6, IL1β, and DAS28, was not different between the new CME group and the remaining RA patients. Further, we compared the new CME group with CMR group, which accumulated CVR factors and had the highest eCVR (Figure 1). The baseline parameters of the patients with new CME were similar to the CMR group with respect to eCVR and also sRAGE (Figure 3B). We observed no differences in other cardiometabolic and inflammatory parameters between those groups.

## Discussion

In the present study we show that low serum levels of sRAGE are significantly associated with previous history and with new imminent cardiometabolic events in female RA patients. In both cases, this corresponded to higher estimation of CVR in the patients with low sRAGE. This low sRAGE was largely attributed to adverse metabolic parameters rather than signs of inflammation. We observed high fasting plasma glucose, and overweight to be the major contributors to CVD risk in younger sRAGE^lo^ group. Also, 5-year follow up showed that the patients with new CME had remarkably low sRAGE levels and reach the level of CMR group in eCVR. The patients with new CME displayed significant accumulation of unfavorable metabolic factors combining high plasma glucose with overweight.

Recently, Dozio et al. suggested circulating sRAGE as an early marker of cardiometabolic disease. They showed that healthy obese women presented lower sRAGE levels than normal-weight women, and found inverse association between sRAGE levels with BMI, total fat mass, and visceral fat in the epicardial region (32). In another study, investigating healthy subjects from the general population with no T2D, CVD, hypertension, or treatment for hyperlipidemia, the authors found that BMI and waist circumference were inversely associated with sRAGE in women (33). Consistent with above findings in healthy women, we observed a significant association between low sRAGE levels with total body fat and eCVR in the patients below 50 years. Similarly, patients with new CME had low sRAGE and displayed significantly increased plasma glucose levels and body fat content compared to both sRAGE^lo^ in sRAGE^hi^ groups. These findings suggest that low sRAGE reflects a metabolic misbalance prior to imminent clinical CME.

In our cohort, we have analyzed the total levels of circulating sRAGE, which exists in two main isoforms—a soluble RAGE cleaved from the membrane-bound full-length RAGE by proteases (34–36), and an endogenously secreted RAGE produced by alternative splicing (15). While cleaved RAGE has a strong association with inflammation markers, endogenously secreted RAGE remains constant among age groups in the healthy population and reflects metabolic disturbances related to obesity and insulin resistance (29). In our sRAGE^hi^ group, sRAGE levels were primarily associated with inflammation including disease activity and IL6. In contrary, in sRAGE^lo^ group, sRAGE had a negative correlation with BMI, eCVR and age, reflecting metabolic disturbance rather than inflammation. What could explain these seemingly controversial associations?

Under physiological conditions, the cell surface RAGE has relatively low expression in non-inflamed tissues (37) whereas during inflammation, it is up-regulated responding to ligand exposure (1). In active RA, a plethora of inflammatory ligands for RAGE are present, both in the synovium (38–40) as well as in the circulation (41, 42) thereby modulating the expression of cell-bound RAGE. In our sRAGE^hi^ patient group, but not in sRAGE^lo^ group, we observed a positive correlation between markers of inflammation. In fact, the more inflammatory ligands for RAGE in the surrounding milieu, the higher expected density of the cell-bound receptor, which predisposes to increased production of sRAGE by cleavage and sRAGE levels are probably a simple reflection of RAGE production in tissues.

Besides inflammation, another molecular explanation for decreased sRAGE is conceivable. Hyperglycemia leads to increased non-enzymatic glycation of proteins, i.e., production of AGEs. Soluble RAGE binds AGEs without activating cellular pathways and functions as a decoy to AGEs, increasing its consumption and decreasing detectable circulating level. On the other hand, binding up AGEs leads to blocking of AGE-RAGE signaling, reduces the positive feedback loop for RAGE up-regulation and thereby potentially limits enzymatic cleavage of RAGE. This explanation is well-applicable for the patients of CMRG and new CME groups, both recognized by low sRAGE and high plasma glucose level.

Of importance, the levels of circulating sRAGE could be affected by several drugs. The effect of treatment for hypertension and hyperlipidemia (22, 43) as well as DMARD treatment with methotrexate (23) has been shown to modulate sRAGE levels in several studies. However, in our cohort, we did not find any differences in the level of sRAGE between groups either treated or not with statins, DMARDs or antihypertensive drugs Supplementary Table 1.

Our study has certain limitations, which need to be taken into account. Firstly, the study had a cross-sectional design, although the patients were clinically followed up for 5 years with respect to cardiometabolic events. A structured consecutive blood sampling during the follow up period would have probably rendered more clear-cut results. However, data from the community-based atherosclerosis risk study ARIC, which measured sRAGE levels with 3 years apart, suggested that sRAGE concentrations within individual subjects are relatively stable. Thus, a single measure could be valuable to evaluate the long-term CV risk (44). Secondly, as discussed above, we have analyzed the total amount of sRAGE and therefore the study does not permit any conclusions with regards to sRAGE isoforms and their relations to CVR in RA women. This question needs to be addressed in future studies.

Taken together, this study shows that low sRAGE reflected higher CV risk in female RA patients. It was associated with previous history and with new forthcoming cardiometabolic events. The study also emphasizes metabolic misbalance behind low sRAGE in female RA patients and puts it forward as a useful biomarker to monitor cardiometabolic health in RA patients.

## Data Availability Statement

The original contributions presented in the study are included in the article/Supplementary Material, further inquiries can be directed to the corresponding author/s.

## Ethics Statement

The studies involving human participants were reviewed and approved by the Ethical Review Board of Gothenburg with permission code 659-2011. All methods used in this study were carried out in accordance with relevant Swedish guidelines and regulations and following the Good Clinical Practice. The informed written consent was obtained from all subjects prior to enrolment in the study.

## Author Contributions

MB and RP: conceptualization, funding acquisition, and resources. MN, LL, SS, and ME: data curation. MN, ME, MB, and RP: formal analysis. MN, LL, SS, ME, and KA: investigation. MN and MB: methodology. SS and MB: project administration. ME: software. RP: supervision. MN, MB, and RP: validation. MN, ME, and RP: visualization. MN: writing—original draft. MN, LL, SS, ME, KA, MB, and RP: writing—review and editing. All authors contributed to the article and approved the submitted version.

## Conflict of Interest

The authors declare that the research was conducted in the absence of any commercial or financial relationships that could be construed as a potential conflict of interest.
