# Supplementary material for: Low Soluble Receptor for Advanced Glycation End Products Precedes and Predicts Cardiometabolic Events in Women With Rheumatoid Arthritis
Source: Front Med (Lausanne). 2021 Jan 28;7:594622. doi: 10.3389/fmed.2020.594622 (PMC7876441; doi:10.3389/fmed.2020.594622)

Supplementary Material

# Supplementary Table

**The frequency of different medication in RA women in sRAGE^lo^ and sRAGE^hi^ group.**

| Medication | sRAGE^hi^  (n=73) | sRAGE^lo^  (n=98) | p-value | Odds  Ratio |
| --- | --- | --- | --- | --- |
| Biologic DMARDs | 21 | 35 | 0.43 | 0.73 |
| MTX monotherapy | 33 | 46 | 0.87 | 0.93 |
| TNF-inhibitors | 15 | 25 | 0.47 | 0.75 |
| NSAIDs | 39 | 60 | 0.35 | 0.73 |
| prednisolone | 9 | 10 | 0.84 | 1.24 |
| Lipid lowering therapy | 1 | 3 | 0.86 | 0.44 |
| ACE-inhibitor | 1 | 5 | 0.38 | 0.26 |

The difference is calculated as odds ratio (OR) with 95% confidence interval (CI). The p-values are obtained by Fisher's exact test.

ACE– angiotensin converting enzyme; DMARD – biological disease modifying anti rheumatic drug; MTX– methotrexate; NSAID– none steroidal anti-inflammatory drug; sRAGE– soluble receptor for advanced glycation end products; TNF–Tumor necrosis factor;

# Supplementary Figure 1

**The correlation analysis between sRAGE and eCVR.**

The univariate correlation analysis in un-dichotomized RA cohort (A) and in patients dichotomized into sRAGE^lo^ and sRAGE^hi^ groups (B) with observed bi-directional correlation profile between sRAGE and eCVR. The correlation coefficient and p-value are indicated on the figures for respective sRAGE^lo^ and sRAGE^hi^ groups.

**A.**


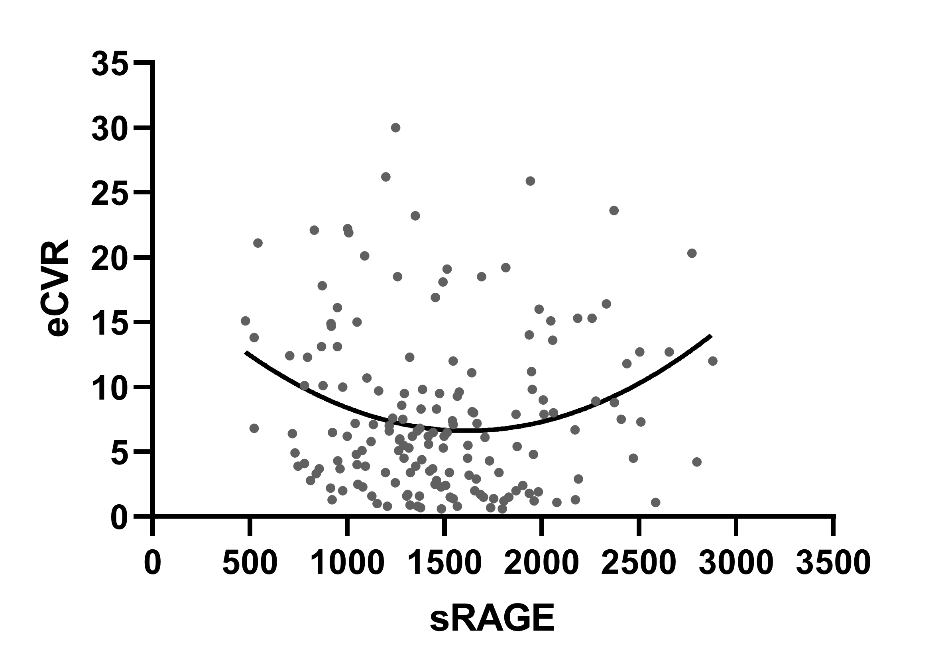


**B.**


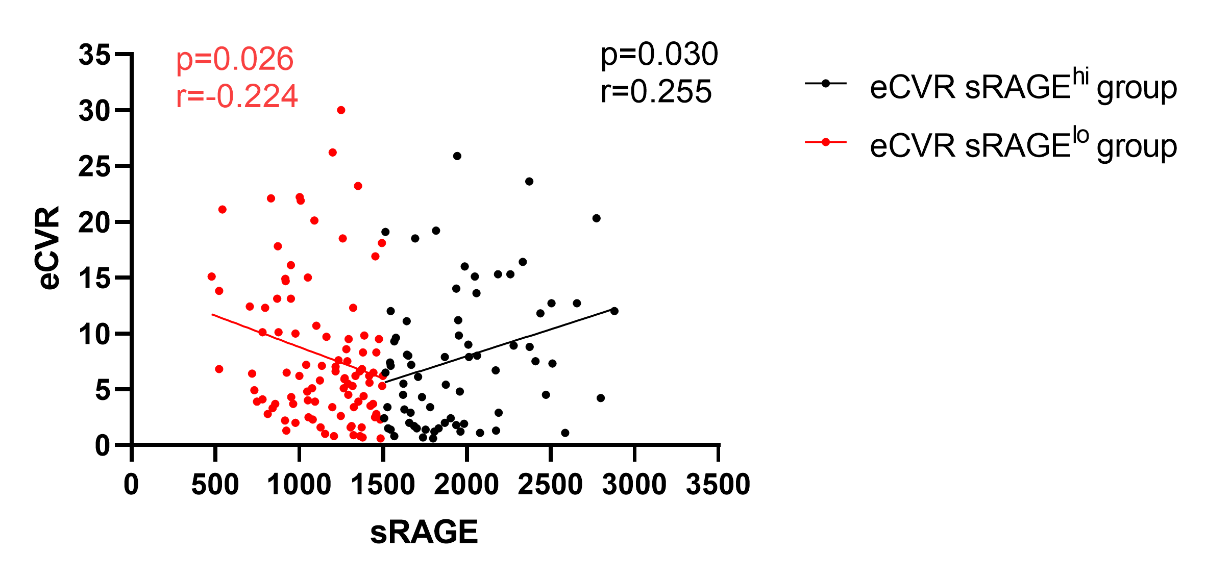

Supplement: Supplementary file 1 [file Data_Sheet_1.docx]
